# Supplementary material for: Chromosomal barcodes for simultaneous tracking of near-isogenic bacterial strains in plant microbiota
Source: Nat Microbiol. 2024 Mar 19;9(4):1117–29. doi: 10.1038/s41564-024-01619-8 (PMC10994850; doi:10.1038/s41564-024-01619-8)
Supplement: Supplementary file 1 — Supplementary text. [file 41564_2024_1619_MOESM1_ESM.pdf]

nature microbiology

Resource https://doi.org/10.1038/s41564-024-01619-8

Chromosomal barcodes for simultaneous tracking of near-isogenic bacterial strains in plant microbiota

In the format provided by the authors and unedited

Principle of spike-based normalization for microbial load estimates

Conventional profiling of plant-associated microbial communities does not provide information on microbial load (Fig. 2a). Thus, two samples with the same ratios of bacteria but different amounts of each strain appear identical when calculating relative abundances. As the sample DNA consists of >99% plant DNA, the spike to plant DNA concentrations can be adjusted. This allows normalization of *16S rRNA* reads using the spike reads to compare strain abundances between samples.

Barcode DNA tag-specific amplification efficiencies using diverse tag-flanking primers

To test whether barcode DNA tag-specific amplification efficiencies were present when using other, non-*16S*-specific primers, we performed qPCR with dilution series of genomic DNA from MoBacTag-labeled wild-type WCS358, WCS358:pqqF and WCS358:cyoB using barcode-specific, plant *ITS*-specific and inter-gene chromosome-specific primers. The amplification efficiencies of the inter-gene chromosome-specific sequence were comparable for all three strains, while we observed tag-specific amplification efficiencies with both the barcode-specific and plant *ITS*-specific primers (Extended Data Fig. 3).

Response of a root-associated community to a MoBacTag-labeled commensal

To investigate whether a chromosomally integrated MoBacTag alters bacterial community structure in root or peat matrix compartments, microbiota reconstitution experiments were performed using gnotobiotic *Arabidopsis* seedlings grown in Jiffy-based gnotopots with defined (synthetic) bacterial communities (SynComs)[1]. A taxonomically diverse SynCom consisting of 15 *Arabidopsis* root-derived bacteria from the *At*-R-SPHERE culture collection was co-inoculated with *Pseudomonas capeferrum* WCS358 wild-type or the MoBacTag-labeled variant WCS358:BC (SynCom modified from Wippel, et al. [2]). As all SynCom members were distinguishable on the basis of their endogenous *V5–V7 16S rRNA* sequences, Principal Coordinate Analyses (PCoA) of Bray–Curtis dissimilarities of *V5–V7 16S rRNA* reads normalized to spike reads were performed to assess potential differences in bacterial community composition in the presence of *P. capeferrum* WCS358 wild-type or its MoBacTag-labeled variant. Most of the variation was explained by compartments, separating root and peat matrix-associated communities (64% of variation in the first component; Extended Data Fig.

5a-c), confirming that live plant roots exert a major influence on SynCom composition. We expected significant differences in root- and peat matrix-derived bacterial communities in the presence of wild-type *P. capeferrum* WCS358 or the barcoded variant WCS358:BC since the MoBacTag is treated as an additional strain/*V5–V7 16S rRNA* sequence (Extended Data Fig. 5a). Indeed, the differences in community composition decreased after *in silico* depletion of MoBacTag- and WCS358 *16S rRNA*-specific reads (*p*-values > 0.01 after *in silico* depletion of reads; Extended Data Fig. 5b,c). Thus, the bacterial community harboring a MoBacTag-labeled WCS358 strain is very similar to the community containing the untagged WCS358 wild-type strain.

Consistent with the Bray–Curtis dissimilarity analyses, the abundance of individual bacterial community members in root and peat matrix compartments were very similar in communities formed in the presence of untagged WCS358 or the MoBacTag-labeled variant WCS358:BC (Extended Data Fig. 5d,e). A few SynCom members diverged in abundance depending on the presence of unlabeled WCS358 or labeled WCS358:BC, such as *Pseudomonas* spp. R68 in the root compartment (Extended Data Fig. 5d) or *Streptomyces* spp. R1310 and Xanthomonadales R480 in the peat matrix compartment (Extended Data Fig. 5e), but these differences were not consistent across independent replicates (Extended Data Fig. 6). Accordingly, the observed differences are likely due to intrinsic stochastic effects during the establishment of bacterial communities as supported by the analysis of individual, independent experiments (Extended Data Fig. 6) and do not result from the gentamicin resistance cassette introduced together with the MoBacTag fragment (Fig. 1b). In conclusion, the chromosomally integrated MoBacTag fragment in WCS358 had little influence on the establishment of 15-member bacterial communities in both the root and peat matrix compartments.

Guide to eliminate the antibiotic resistance from the MoBacTag using *FRT* sites[3,4]

MoBacTag-encoded antibiotic resistance cassettes are flanked by *FRT* sites and can therefore be excised from the chromosome using FLP recombinase.

Step A: Transformation of *Flp* recombinase coding plasmid pFLP3 into MoBacTag-labeled strain:

- Transform pFLP3 plasmid into the MoBacTag-labeled strain using a suitable transformation protocol.
- Select transformants by plating dilution series of the transformation on cultivation medium supplemented with Tetracycline 10 $\mu g.ml^{-1}$ or Ampicillin 100 $\mu g.ml^{-1}$.
- Incubate plates for 2-5 days at bacterial culture conditions.

Step B: Check for the antibiotic susceptibility and validation by PCR-based genotyping

- Inoculate single colonies in culture medium supplemented with either antibiotic for which the corresponding resistance cassette has been putatively eliminated and medium supplemented with Tetracycline 10 $\mu g.ml^{-1}$ or Ampicillin 100 $\mu g.ml^{-1}$.
- Test colonies only growing on medium supplemented with Tetracycline 10 $\mu g.ml^{-1}$ or Ampicillin 100 $\mu g.ml^{-1}$ by PCR-based genotyping:
    - Extract DNA and perform PCR as described in "Labeling of bacterial strains with MoBacTags using mini Tn7 system".
    - Primers should be designed to bind to the regions flanking MoBacTag-encoded *FRT* sites.

Step C: Curation of the pFLP3 plasmid

- pFLP3 encodes *sacB* derived from *B. subtilis* confering sucrose sensitivity.
- Plate PCR-validated colonies on cultivation medium supplemented with 5% of sucrose.
- Test colonies for loss of pFLP3 by PCR-based genotyping using pFLP3-specific primers.

References

1 Kremer, J. M. *et al.* Peat-based gnotobiotic plant growth systems for Arabidopsis microbiome research. *Nat Protoc* **16**, 2450-2470 (2021). https://doi.org:10.1038/s41596-021-00504-6

2 Wippel, K. *et al.* Host preference and invasiveness of commensal bacteria in the Lotus and Arabidopsis root microbiota. *Nat Microbiol* **6**, 1150-1162 (2021). https://doi.org:10.1038/s41564-021-00941-9

3 Choi, K. H. & Schweizer, H. P. mini-Tn7 insertion in bacteria with single attTn7 sites: example Pseudomonas aeruginosa. *Nat Protoc* **1**, 153-161 (2006). https://doi.org:10.1038/nprot.2006.24

4 Choi, K. H. *et al.* A Tn7-based broad-range bacterial cloning and expression system. *Nat Methods* **2**, 443-448 (2005). https://doi.org:10.1038/nmeth765
