# Supplementary material for: Chromosomal barcodes for simultaneous tracking of near-isogenic bacterial strains in plant microbiota
Source: Nat Microbiol. 2024 Mar 19;9(4):1117–29. doi: 10.1038/s41564-024-01619-8 (PMC10994850; doi:10.1038/s41564-024-01619-8)
Supplement: Supplementary file 2 — Reporting Summary [file 41564_2024_1619_MOESM2_ESM.pdf]

nature portfolio

Corresponding author(s): Paul Schulze-Lefert

Last updated by author(s): Dec 31, 2023

Reporting Summary

Nature Portfolio wishes to improve the reproducibility of the work that we publish. This form provides structure for consistency and transparency in reporting. For further information on Nature Portfolio policies, see our Editorial Policies and the Editorial Policy Checklist.

Statistics

For all statistical analyses, confirm that the following items are present in the figure legend, table legend, main text, or Methods section.

n/a	Confirmed	
☐	☒	The exact sample size (n) for each experimental group/condition, given as a discrete number and unit of measurement
☐	☒	A statement on whether measurements were taken from distinct samples or whether the same sample was measured repeatedly
☐	☒	The statistical test(s) used AND whether they are one- or two-sided<br>*Only common tests should be described solely by name; describe more complex techniques in the Methods section.*
☐	☒	A description of all covariates tested
☐	☒	A description of any assumptions or corrections, such as tests of normality and adjustment for multiple comparisons
☐	☒	A full description of the statistical parameters including central tendency (e.g. means) or other basic estimates (e.g. regression coefficient) AND variation (e.g. standard deviation) or associated estimates of uncertainty (e.g. confidence intervals)
☐	☒	For null hypothesis testing, the test statistic (e.g. F, t, r) with confidence intervals, effect sizes, degrees of freedom and P value noted<br>*Give P values as exact values whenever suitable.*
☒	☐	For Bayesian analysis, information on the choice of priors and Markov chain Monte Carlo settings
☐	☒	For hierarchical and complex designs, identification of the appropriate level for tests and full reporting of outcomes
☒	☐	Estimates of effect sizes (e.g. Cohen's d, Pearson's r), indicating how they were calculated

Our web collection on statistics for biologists contains articles on many of the points above.

Software and code

Policy information about availability of computer code

Data collection	16S amplicon data were collected using a MiSeq Illumina sequencer. Reads were demultiplexed with QIIME, merged with FLASH2, cleaned with USEARCH and the amplicon sequencing variant table was generated with Rbec. The genomes were sequenced with a Sequel IIe Pacific Biosciences sequencer and assembled with Hifiasm.
Data analysis	QIIME v2 2021.2<br>FLASH2 V.2.2.00<br>USEARCH v10.0.240<br>Rbec v1.8.0<br>R v4.0.0<br>R package vegan v2.6-4<br>R package ggplot2 v3.4.4<br>Hifiasm v0.16.1<br>Mauve v2015-02-25<br>gepard v2.1.0<br>WebLogo<br>Costumized code is available here: https://github.com/thouinjulien/MoBacTag

For manuscripts utilizing custom algorithms or software that are central to the research but not yet described in published literature, software must be made available to editors and reviewers. We strongly encourage code deposition in a community repository (e.g. GitHub). See the Nature Portfolio guidelines for submitting code & software for further information.

Data

Policy information about availability of data

All manuscripts must include a data availability statement. This statement should provide the following information, where applicable:

- Accession codes, unique identifiers, or web links for publicly available datasets
- A description of any restrictions on data availability
- For clinical datasets or third party data, please ensure that the statement adheres to our policy

Raw 16S rRNA amplicon and genome reads are deposited in the European Nucleotide Archive (ENA) under the accession number PRJEB61076. For the A. thaliana Col-0 sequence the TAIR9 assembly was used (www.arabidopsis.org). Bacterial At-R-SPHERE 16S sequences were retrieved from the website https://www.at-sphere.com.

Research involving human participants, their data, or biological material

Policy information about studies with human participants or human data. See also policy information about sex, gender (identity/presentation), and sexual orientation and race, ethnicity and racism.

Reporting on sex and gender	NA
Reporting on race, ethnicity, or other socially relevant groupings	NA
Population characteristics	NA
Recruitment	NA
Ethics oversight	NA

Note that full information on the approval of the study protocol must also be provided in the manuscript.

Field-specific reporting

Please select the one below that is the best fit for your research. If you are not sure, read the appropriate sections before making your selection.

☒ Life sciences ☐ Behavioural & social sciences ☐ Ecological, evolutionary & environmental sciences

For a reference copy of the document with all sections, see nature.com/documents/nr-reporting-summary-flat.pdf

Life sciences study design

All studies must disclose on these points even when the disclosure is negative.

Sample size	Previous experiments using the same gnotobiotic system were used as reference to determine sample size and to allow confident statistical analyses (e.g. Wippel et al., 2021. Nature Microbiology; Kremer et al., 2021. Nature Protocols). For each figure, the sample size is indicated as "n" in the corresponding figure legend. No statistical methods were used to determine sample size.
Data exclusions	No data were excluded from the analyses.
Replication	The gnotobiotic experiment (Figure 4 to 6) was performed with 3 independent preparations of the corresponding SynComs (biological replicates) and with at least 8 technical replicates of planted pots for each independent SynCom preparations.<br>The molecular biology to validate the oligonucleotides from Figure 2 was performed with at least 15 technical replicates.<br>The inoculation of MoBacTag-labeled Rhizobia strains into native soils was performed with 2 independent experiments and with 8 technical replicates per experiment per soil type. No attempt of replication was excluded from the analysis.
Randomization	Position of boxes with planted pots was randomized in the growth chamber during the course of the experiments.
Blinding	The samples were harvested and processed by multiple researchers. Each SynCom condition or compartment were processed by every researcher.<br>After setting-up the experiment, boxes and samples were labeled with unique numbers to blind the researcher during the harvesting and processing.

Reporting for specific materials, systems and methods

We require information from authors about some types of materials, experimental systems and methods used in many studies. Here, indicate whether each material, system or method listed is relevant to your study. If you are not sure if a list item applies to your research, read the appropriate section before selecting a response.

Materials & experimental systems

n/a	Involved in the study	
☒	☐	Antibodies
☒	☐	Eukaryotic cell lines
☒	☐	Palaeontology and archaeology
☒	☐	Animals and other organisms
☒	☐	Clinical data
☒	☐	Dual use research of concern
☐	☒	Plants

Methods

n/a	Involved in the study	
☒	☐	ChIP-seq
☒	☐	Flow cytometry
☒	☐	MRI-based neuroimaging
